# The Health Belief Model Predicts Intention to Receive the COVID-19 Vaccine in Saudi Arabia: Results from a Cross-Sectional Survey

**DOI:** 10.3390/vaccines9080864

**Published:** 2021-08-05

**Authors:** Ilias Mahmud, Russell Kabir, Muhammad Aziz Rahman, Angi Alradie-Mohamed, Divya Vinnakota, Abdulrahman Al-Mohaimeed

**Affiliations:** 1 Department of Public Health, College of Public Health and Health Informatics, Qassim University, Al Bukairiyah 51941, Saudi Arabia; 2 School of Allied Health, Faculty of Health, Education, Medicine and Social Care, Anglia Ruskin University, Chelmsford, Essex CM1 1SQ, UK; russell.kabir@aru.ac.uk (R.K.); drdivya0424@gmail.com (D.V.); 3 School of Health, Federation University Australia, Berwick, VIC 3806, Australia; ma.rahman@federation.edu.au; 4 Australia Institute for Primary Care and Ageing (AIPCA), La Trobe University, Melbourne, VIC 3086, Australia; 5 Faculty of Public Health, Universitas Airlangga, Surabaya 60115, Indonesia; 6 United Arab Emirates University, Al Ain P.O. Box 15551, United Arab Emirates; Ngmotsim@gmail.com; 7 Department of Family and Community Medicine, College of Medicine, Qassim University, Buraydah 52571, Saudi Arabia

**Keywords:** COVID-19, SARS-CoV-2, vaccine hesitancy, health belief model, Saudi Arabia

## Abstract

We examined the intention and predictors of accepting the COVID-19 vaccine in Saudi Arabia. We conducted a nation-wide, cross-sectional online survey between February and March 2021. A total of 1387 people (≥18 years) participated. Only 27.3% adults had a definite and 30.2% had a probable vaccination intent; 26.8% and 15.6% had a probable and definite negative vaccination intent. Older people (≥50 years) (*p <* 0.01), healthcare workers/professionals (*p* < 0.001), and those who received flu vaccine (*p* < 0.001) were more likely to have a positive intent. People from Riyadh were less likely to receive the vaccine (*p* < 0.05). Among the health belief model constructs, perceived susceptibility to and severity of COVID-19 (*p* < 0.001), and perceived benefit of the vaccine (*p* < 0.001) were positively associated with vaccination intent, whereas perceived barriers had a negative association (*p* < 0.001). Individuals were more likely to receive the vaccine after obtaining complete information (*p* < 0.001) and when the vaccine uptake would be more common amongst the public (*p* < 0.001).

## 1. Introduction

Coronavirus disease 2019 (COVID-19) is caused by the Severe Acute Respiratory Syndrome Coronavirus 2 (SARS-CoV-2) first reported in Wuhan, China, in December 2019 [1,2]. Within a few months it dispersed widely across the globe, causing severe humanitarian and economic burden, and overwhelmed health systems globally. As of 12 May 2021, the World Health Organization (WHO) reported over 164.5 million confirmed cases of COVID-19, including about 3.5 million deaths, globally [3]. In the Kingdom of Saudi Arabia (KSA), the first COVID-19 case was identified on 2 March 2020. The KSA took early precautionary responses against the pandemic. The authorities enforced several timely and judicious preventive measures, including lockdown, to limit the spread of the virus [4]. These actions were believed to decrease the number of cases, fatality, and minimized economic burden in comparison with other countries [5]. Despite these efforts, by 20 May 2021 the KSA recorded 436,240 COVID-19 cases including 7201 deaths [3].

The lack of proven treatment [6], severe burden of COVID-19 infection and its fast spread generated an urgent need of a vaccine against COVID-19, leading to an unprecedented scale and speed in the effort to develop the COVID-19 vaccine; the development hit a record by entering human clinical trial in March 2020 [7]. By April 2020, more than 100 vaccine candidates were developed across different countries using different methods; five of which were in the clinical evaluation stage, and 73 at exploratory or pre-clinical stage [7,8]. As of 12 November 2020, the number of candidate vaccines in preclinical evaluation grew to 164, and those in clinical evaluation became 48 vaccines with four vaccines cleared for phase three trials [9]. By 7 May 2021, five vaccines against COVID-19 received approval from the WHO to roll out globally. These vaccines were the Pfizer/BioNTech vaccine (USA), two AstraZeneca/Oxford vaccines produced by AstraZeneca-SKBio (Republic of Korea) and the Serum Institute of India, COVID-19 vaccine Ad26.COV2.S developed by Janssen (Johnson & Johnson), and the Sinopharm COVID-19 vaccine produced by Beijing Bio-Institute of Biological Products Co Ltd., subsidiary of China National Biotec Group [10]. During the first quarter of 2021, the Saudi Ministry of Health was using two COVID-19 vaccines—Pfizer/BioNTech BNT162b2 and AstraZeneca/Oxford AZD1222—to inoculate people in the Kingdom [11]. For the COVID-19 vaccine to be effective in containing the epidemic within a country without other measures like social distancing, a big percentage of the population needs to be vaccinated [12,13].

There were differences in the acceptance rate of COVID-19 vaccine in different countries—less than 55% in Russia to 90% in China [14]. In the KSA, in December 2020, 50.5% of health workers [15] and 48% of the general population [16] expressed their intent to receive the COVID-19 vaccine. While another study conducted in January–February 2021 reported that only 20.3% of the study participants had registered to receive the vaccine [17]. According to a study conducted in the USA, willingness to receive COVID-19 vaccine declined quickly from 71% in April to 53.6% in October 2020, whereas the proportion unwilling to receive the vaccine increased from 18% to 32% in the same period. Most of those unwilling to be vaccinated feared the side effects and long-lasting health complications in addition to being uncertain of the vaccine benefits [18], not to mention that misinformation, rumours, and conspiracy theories about COVID-19 negatively affected the willingness of the population to be vaccinated [19,20].

The WHO identified vaccine hesitancy as a leading threat to global health. Vaccine hesitancy could be due to inconvenience in accessing vaccines, complacency, or lack of trust [21]. In addition, different unmeasurable influence/power could make the issue more complex that depends on context, time, place, and type of vaccine [22]. A multi-country study on COVID-19 vaccine hesitancy [14] reported that a positive attitude towards the COVID-19 vaccine was associated with higher levels of education, income, a medium to high number of cases and fatality rates, and trust in the respective government. In KSA, willingness to receive the COVID-19 vaccine, even when it was not available, was significantly associated with being >45 years old and married [23].

The Health Belief Model (HBM) is a conceptual framework that has been used to explain, predict, and influence behaviours of individuals or groups in relation to health issues. This model explains that actions related to health issues need the existence of sufficient motivation (e.g., illness or health concern), a perceived threat, the belief of a serious health problem/complication caused by that illness, perceived benefits, the belief that abiding by health recommendations would be beneficial in reducing the perceived threats, and that the benefits outweigh the costs [24]. The HBM was used by several studies to predict the intention to receive vaccines. For example, it was used to predict uptake of the influenza vaccine [25,26,27,28,29], parents’ intention regarding vaccination of their children [30], and intention of females to receive HPV vaccine [31]. In the KSA, the hesitancy in receiving the influenza vaccine was associated with the belief that it was not beneficial, people had fear of side effects, and they had a lack of perceived threat from influenza [32]. Recently, this model was used to predict COVID-19 vaccination intention in different countries [33,34,35,36].

The KSA imposed phased restriction measures to contain the COVID-19 epidemic with the aim to gradually and cautiously return to normal life [4]. To achieve that goal, the country needs to inoculate most of the population. A study in the USA suggested that with 80% vaccine efficacy and 10% mask usage, 90% vaccine coverage is needed to contain the epidemic in the USA. The coverage could be decreased to 82% if mask compliance increased to 50% of the population [13]. Therefore, it was of paramount importance to study the current level of COVID-19 vaccine acceptance and identify the influencing factors to assist the government and public health officials in addressing vaccine hesitancy and plan accordingly to improve vaccine uptake in KSA. In this context, this study examined the intention to receive the COVID-19 vaccine, and whether socio-demographic and HBM predicted such intention among the adults in the KSA.

## 2. Methods

We conducted a nationwide online cross-sectional survey in the first quarter of 2021. Study population included adult (≥18 years) citizens or residents of Saudi Arabia. To disseminate our online questionnaire, we used all commonly used social media platforms in Saudi Arabia. In addition, we used our professional online groups and email networks to disseminate the questionnaire. Individuals completing the questionnaire were requested to forward the survey link to their networks. The minimum required sample size for this study was 384. Sample size was calculated using the Epi Info^TM^ 7. For sample size calculation, we considered the following conditions: 50% expected intention frequency, 80% power, and 5% acceptable margin of error.

Our questionnaire included questions on socio-demographic variables, health status, COVID-19 experience, intention to receive a COVID-19 vaccine and HBM constructs related to COVID-19 vaccination. Socio-demographic questions included age, gender, ethnicity, religion, marital status, education, and occupation. To assess health status, we collected information on diagnosis of chronic diseases. To assess participants’ COVID-19 experience, we collected information on COVID-19 infection among participants, their family members, relatives, friends, neighbours, or colleagues. Participants’ intention to receive a COVID-19 vaccine was assessed using a one-item question (if a vaccine against COVID-19 infection is available for you, would you take it?) on a five-point Likert type scale (‘No, definitely not’ to ‘Yes, definitely’).

We developed the questionnaire reviewing relevant literature. To ensure the relevance and clarity of the questions, the content validity of the questionnaire was assessed by a panel of public health scientists in KSA. In addition, the questionnaire was pre-tested among a group of university students. 

To assess the HBM constructs we asked three questions to assess perceived susceptibility to COVID-19 infection, three questions to assess perceived severity of COVID-19 infection, two questions to assess perceived benefits of a COVID-19 vaccine, five questions to assess perceived barriers to getting a vaccination against COVID-19 and two questions to assess cues to action. We used a simplified response options—agree/disagree—since we did an online self-administered survey. 

Data were analysed using STATA v.12 (StataCorp LLC, TX, USA). All the study variables were categorical. At first, we conducted descriptive analyses to show the proportions of the study variables including socio-demographics, chronic diseases, diagnoses of COVID-19, and status of flu vaccination. Then, descriptive analyses were presented to show the proportions of COVID-19 related health beliefs in the category of agree or disagree. The primary study variable of our interest was intention to take COVID-19 vaccine, which collected data in four categories, such as ‘definitely not’, ‘probably not’, ‘definitely yes’, and ‘probably yes’. For analyses, we re-categorised them into dichotomous responses, as ‘definitely or probably yes’ and ‘definitely or probably no’. Then we examined the factors associated with intention to receive COVID-19 vaccine; chi-square tests were used to determine statistically significant association as indicated by *p* value < 0.05, followed by bivariate and multivariate logistic regression to determine the strength of association. Odds Ratio (OR), Adjusted Odds Ratio (AOR) and 95% Confidence Intervals (CIs) were calculated. In the same way, we examined the association between perceived COVID-19 related health beliefs and intention to receive a COVID-19 vaccine. We adjusted gender, age, ethnicity, regions, education, occupation, presence of chronic disease, diagnoses of COVID-19, and status of flu vaccination during multivariate analyses.

Ethical approval was obtained from the Qassim Regional Ethics Committee (IRB number: H-04-Q-001). In addition, approval was obtained from the Saudi Center for Disease Control and Prevention (registration number: 202102011). All participants were informed about the objectives of the study. They were also informed that participating in this study was completely voluntary and was not associated with any benefits or harms. The first page of the online survey form included the informed consent form. 

## 3. Results

A total of 1387 people participated in our study; most of them (61%) were male. One third of the study participants belonged to the age group 18–29 years and the other third belonged to the 30–39 years age group. Most of them were Saudi (86%) and almost half of them were from Riyadh (46%). Most of them had tertiary education (85%) and one in five were healthcare workers/professionals (21%). COVID-19 was diagnosed amongst 15% of the study participants and a quarter of them (25%) reported receiving the flu vaccine every year. More than half of the study participants (58%) intended to receive the COVID-19 vaccine (Table 1).

When COVID-19–related health beliefs were analysed, as shown in Table 2, it was found that the majority of the study participants disagreed on the perceived susceptibility to getting COVID-19. While severity of complications was perceived as very serious by more than half of the study participants (58%), a quarter of them (27%) agreed that they would be very sick if they got COVID-19. Two thirds of the study participants (66%) perceived benefits of getting COVID-19 vaccine. However, the majority of them were concerned about the efficacy and safety/side effects. Most of them (83%) were not concerned of the halal nature of the vaccine. A third of the study participants (32%) intended to receive the vaccine if it would be received by many in the public, but the majority of them intended to receive the vaccine after receiving complete information and if the vaccine did not cause indue problems to the vaccinated people (Table 2).

Table 3 showed unadjusted and adjusted analyses regarding factors associated with intention to receive the COVID-19 vaccine. Unadjusted analyses showed that older people (50 years old and above), people from Qassim, healthcare workers/professionals and those who used to receive the flu vaccine every year were more likely to receive COVID-19 vaccine. However, those who identified themselves as Saudi, people from Riyadh, general workers, students, and unemployed people were less likely to elect to receive the COVID-19 vaccine. Following adjustment of potential confounders, we found that three groups of people were more likely to get COVID-19 vaccine: older people (50 years old and above) (AOR 2.11, 95% CIs 1.38–3.23, *p* < 0.01), healthcare workers/professionals (AOR 2.50, 95% CIs 1.58–3.94, *p* < 0.001), and those who used to receive the flu vaccine every year (AOR 2.63, 95% CIs 1.93–3.58, *p* < 0.001). People from Riyadh were less likely to get the COVID-19 vaccine (AOR 0.72, 95% CIs 0.55–0.96, *p* < 0.05) (Table 3).

Table 4 showed univariate and multivariate analyses for the association between perceived COVID-19–related health beliefs and intention to get the COVID-19 vaccine. People who had more agreement on perceived susceptibility, perceived severity, perceived benefits, and cues to actions were more likely to take COVID-19 vaccine. In addition, people who had more disagreement of the perceived barriers, were more likely to get the COVID-19 vaccine.

## 4. Discussion

This study examined the intention to receive the COVID-19 vaccine in KSA and how the health belief model constructs predicted such intention. We found that 15.6% and 26.8% adults would definitely and probably not accept the vaccine, respectively, whereas a recent study in the UK and USA found that the percentage of respondents who would definitely accept the vaccine to protect themselves from COVID-19 was 54.1%. In comparison, 6.0% of the respondents in the UK and 15% in the US reported that they would definitely not accept a COVID-19 vaccine [37]. That indicated that COVID-19 vaccine hesitancy was higher in the KSA compared to other high-income countries. Our study revealed that younger individuals of age group 18–29 (38.2%) were more vaccine hesitant. Similar findings were reported in Ireland and the UK where individuals belonging to the age group 18–24 years were more hesitant to receive the COVID-19 vaccine [38]. Another study in Jordan and Kuwait stated that the acceptance of the COVID-19 vaccine was higher among males and people with higher educational status [39]; similar findings were reported in our study that the acceptance of vaccine was higher in males (62.0%) and those who had tertiary education (84.7%). Our study also suggested that people who were previously infected with COVID-19 were more likely to receive the vaccine than those who were not infected, which was similar to the findings of other studies in Saudi Arabia [23] and France [40]. COVID-19 did not only affect physical health but also the mental health of people globally. Hence, their experience of suffering from COVID-19 might be the reason for the higher intent to receive the vaccine.

A vast majority of the study participants (66%) believed that chances of getting the infection would reduce after taking the vaccine. Similar results were reported for the COVID-19 vaccine among the general population in Russia [41], in Bangladesh [36], and the residents from Northern Italy [42]. On a positive note, most of the participants who indicated high perceived susceptibility, perceived severity, and perceived benefits were more likely to receive the COVID-19 vaccine. This was in line with other study findings in Malaysia where the health belief model was used to assess COVID-19 vaccine intake among the general population [33]. A study conducted in Hong Kong reported that COVID-19 vaccine intake was significantly associated with perceived severity, perceived benefits of the vaccine, cues to action, self-reported health outcomes, and trust in the healthcare system or vaccine manufacturers [43]. Among the perceived barriers, the major concern among the participants was that the vaccine might not be halal. Similarly, a recent study among Bangladeshi residents revealed that the negative intent was associated with the concern that the vaccine might not be halal [36]. This was due to the fact that most of the participants were practicing Islam and would avoid anything that is not permissible by the religion. Despite that, it was evident that the participants who had disagreement on perceived barriers and constructs of HBM were highly likely to accept the COVID-19 vaccine. This was completely contrary to the study conducted in Hong Kong where no correlation was found between perceived barriers and vaccination intention [43]. Another study states that belief in COVID-19 misinformation significantly reduced willingness to get the vaccine [20]. Therefore, van der Linden et al. (2021) suggested that to combat vaccine misinformation, the public should be immunized against misinformation—a process that could draw on the concept of psychological inoculation [44]. Also, vaccine acceptability would increase once additional information about vaccine safety and efficacy could be made available in the public domain, preferably by a trusted, centralized source of information [45]. This study provided a glimpse of COVID-19 vaccine acceptance among the general population of KSA. The main strength of the study was a larger sample size compared to other similar studies on COVID-19 vaccine hesitancy in the KSA. However, we did not have equal representation from all socio-demographic groups such as age, gender, nationality, and region. The cross-sectional nature of the study indicated that we could not draw any causal relationship between intention to receive the COVID-19 vaccine and the associated factors. In addition, we were unable to rule out the possibility of selection bias since we used online platforms such as social media to recruit study participants.

## 5. Conclusions

This study revealed that COVID-19 vaccine hesitancy was high in the KSA. The HBM constructs also predicted willingness to receive COVID-19 vaccine. Perceived susceptibility to and severity of the COVID-19 vaccine and perceived benefits of the vaccine against COVID-19 were positively associated with COVID-19 vaccination intention, whereas perceived barriers were negatively associated. Individuals were more likely to get the vaccine after obtaining complete information and when vaccination would be more common amongst the public. The KSA aimed to return to normal life gradually and cautiously from the COVID-19 restriction measures. In this context, proper vaccination coverage could help in reducing the infection and subsequent mortality rates due to COIVD-19. Therefore, public health campaigns in the KSA should consider adopting the HBM to promote COVID-19 vaccine in the population.

## Figures and Tables

**Table 1:** Characteristics of the study participants.

Characteristics	*n* (%)
**Total study participants**	**1387**
**Gender**	
Male	848 (61.1)
Female	539 (38.9)
**Age**	
18–29 years	454 (32.7)
30–39 years	456 (32.9)
40–49 years	277 (20.0)
50 years or more	200 (14.4)
**Ethnicity**	
Saudi	1193 (86.0)
Non-Saudi (African)	16 (1.2)
Non-Saudi (Asian)	90 (6.5)
Non-Saudi (European)	3 (0.2)
Non-Saudi (Middle East)	69 (5.0)
Non-Saudi (Others)	16 (1.2)
**Regions**	
Al baha	5 (0.4)
Al jouf	58 (4.2)
Asir	39 (2.8)
Eastern	128 (9.2)
Hail	14 (1.0)
Jazan	10 (0.7)
Madinah	77 (5.6)
Narjan	3 (0.2)
Qassim	390 (28.1)
Riyadh	637 (45.9)
Tabuk	17 (1.2)
The Northern Border	9 (0.6)
**Education**	
Primary or below	10 (0.7)
Secondary	199 (14.3)
Tertiary (college/university)	1178 (84.9)
**Occupation**	
General worker	32 (2.3)
Healthcare workers/professionals	284 (20.5)
Housewife	154 (11.1)
Student	203 (14.6)
Unemployed	121 (8.7)
Other professional/managerial	387 (27.9)
Other	206 (14.9)
**Participant has chronic disease**	
No	1116 (80.5)
Yes	229 (16.5)
**Participant diagnosed with COVID-19**	
No	1179 (85.0)
Yes	208 (15.0)
**Family member diagnosed with COVID-19**	
No	837 (60.3)
Yes	550 (39.7)
**Relative/friend/neighbor/colleague diagnosed with COVID-19**	
No	89 (6.4)
Yes	1298 (93.6)
**Receive flu vaccine every year**	
No	1044 (75.3)
Yes	343 (24.7)
**Intent to receive COVID-19 vaccine**	
Definitely not	217 (15.6)
Probably not	372 (26.8)
Definitely yes	379 (27.3)
Probably yes	419 (30.2)

**Table 2:** COVID-19 related health beliefs in the KSA, February–March 2021.

Perceived COVID-19 Related Health Beliefs	Agree, *n* (%)	Disagree, *n* (%)
**Perceived susceptibility**		
Chance of getting COVID-19 in the future is very high	458 (33.0)	929 (67.0)
Currently, getting COVID-19 is a strong possibility	610 (44.0)	777 (56.0)
**Perceived severity**		
Complications of COVID-19 is very serious	804 (58.0)	583 (42.0)
Will be very sick if I get COVID-19	372 (26.8)	1015 (73.2)
**Perceived benefits**		
Vaccination will decrease my chances of getting COVID-19	915 (66.0)	472 (34.0)
**Perceived barriers**		
Concerned about the efficacy of the vaccination available	785 (56.6)	602 (43.4)
Concerned about the safety/side effects of the vaccination available	900 (64.9)	487 (35.1)
Concerned about the halal nature of the vaccination available	230 (16.6)	1157 (83.4)
**Cues to action**		
Will get vaccine after receiving complete information	1025 (73.9)	362 (26.1)
Will get vaccine if it is received by many in the public	447 (32.2)	940 (67.8)
Will get vaccine if it does not cause undue problems to vaccinated people	870 (62.7)	517 (37.3)

**Table 3:** Factors associated with intention to receive a COVID-19 vaccine in KSA, February–March 2021.

Intention to Receive COVID-19 Vaccine	Definitely or Probably YES, *n* (%)	Definitely or Probably NO, *n* (%)	Unadjusted Analyses			Adjusted Analyses		
			*p*	OR	95% CIs	*p*	AOR	95% CIs
**Total study participants**	**798**	**589**						
**Gender**								
Male	495 (62.0)	353 (59.9)		1			1	
Female	303 (38.0)	236 (40.1)	0.428	0.92	0.74–1.14	0.147	0.81	0.61–1.08
**Age**								
18–29 years	229 (28.7)	225 (38.2)		1			1	
30–39 years	269 (33.7)	187 (31.7)	0.010	1.41	1.09–1.84	0.226	1.22	0.88–1.68
40–49 years	160 (20.1)	117 (19.9)	0.055	1.34	0.99–1.82	0.448	1.15	0.80–1.67
50 years or more	140 (17.5)	60 (10.2)	***<0.001***	***2.29***	***1.61–3.27***	***<0.001***	***2.11***	***1.38–3.23***
**Ethnicity**								
Non-Saudi	136 (17.0)	58 (9.8)		1			1	
Saudi	662 (83.0)	531 (90.2)	***<0.001***	***0.53***	***0.38–0.74***	0.644	0.91	0.61–1.36
**Regions**								
Riyadh	327 (41.0)	310 (52.6)	***<0.001***	***0.63***	***0.50–0.77***	***0.025***	***0.72***	***0.55–0.96***
Qassim	249 (31.2)	141 (23.9)	***0.003***	***1.44***	***1.13–1.83***	0.709	0.94	0.68–1.30
**Education**								
Primary or below	5 (0.6)	5 (0.8)		1			1	
Secondary	117 (14.7)	82 (13.9)	0.584	1.43	0.40–5.09	0.477	1.64	0.42–6.36
Tertiary (college/university)	676 (84.7)	502 (85.2)	0.639	1.35	0.39–4.68	0.735	1.26	0.33–4.84
**Occupation**								
General worker	11 (1.4)	21 (3.6)	***0.007***	***0.38***	***0.18–0.79***	0.134	0.54	0.24–1.21
Healthcare workers/professionals	222 (27.8)	62 (10.5)	***<0.001***	***3.28***	***2.41–4.45***	***<0.001***	***2.50***	***1.58–3.94***
Housewife	81 (10.2)	73 (12.4)	0.189	0.80	0.57–1.12	0.261	1.33	0.81–2.20
Student	98 (12.3)	105 (17.8)	***0.004***	***0.65***	***0.48–0.87***	0.582	1.14	0.71–1.82
Unemployed	53 (6.6)	68 (11.5)	***0.002***	***0.55***	***0.37–0.79***	0.471	0.84	0.51–1.36
Other professional/managerial	220 (27.6)	167 (28.4)	0.550	0.96	0.76–1.22	0.233	1.25	0.87–1.79
Other	113 (14.2)	93 (15.8)	0.399	0.88	0.65–1.18	NA	NA	NA
**Participant has chronic disease**								
No	654 (82.0)	504 (85.6)		1			1	
Yes	144 (18.0)	85 (14.4)	0.073	1.31	0.97–1.75	0.782	1.05	0.76–1.45
**Participant diagnosed with COVID-19**								
No	667 (83.6)	512 (86.9)		1			1	
Yes	131 (16.4)	77 (13.1)	0.085	1.31	0.96–1.77	0.237	1.23	0.87–1.74
**Family member diagnosed with COVID-19**								
No	490 (61.4)	347 (58.9)		1			1	
Yes	308 (38.6)	242 (41.1)	0.349	0.90	0.73–1.12	0.332	0.88	0.69–1.14
**Relative/friend/neighbor/colleague diagnosed with COVID-19**								
No	52 (6.5)	37 (6.3)		1			1	
Yes	746 (93.5)	552 (93.7)	0.860	0.96	0.62–1.49	0.300	1.30	0.79–2.13
**Receive flu vaccine every year**								
No	528 (66.2)	516 (87.6)		1			1	
Yes	270 (33.8)	73 (12.4)	***<0.001***	***3.61***	***2.72–4.81***	***<0.001***	***2.63***	***1.93–3.58***

OR: Odds Ratio, AOR: Adjusted Odds Ratio, 95% CIs: 95% Confidence Intervals. Adjusted for: Gender, age, ethnicity, regions, education, occupation, chronic disease, diagnosis of COVID-19, receipt of flu vaccine.

**Table 4:** Association between COVID-19 related health beliefs and intention to receive a COVID-19 vaccine in the KSA, February–March 2021.

Perceived COVID-19 Related Health Beliefs	Definitely or Probably YES, *n* (%)	Definitely or Probably NO, *n* (%)	Unadjusted Analyses			Adjusted Analyses		
			*p*	OR	95% CIs	*p*	AOR	95% CIs
**Total study participants**	**798**	**589**						
**Perceived susceptibility**								
Chance of getting COVID-19 in the future is very high	466 (58.4)	463 (78.6)		1			1	
Disagree	332 (41.6)	126 (21.4)	***<0.001***	***2.62***	***2.06–3.33***	***<0.001***	***2.16***	***1.65–2.82***
Agree								
Currently, getting COVID-19 is a strong possibility								
Disagree	390 (48.9)	387 (65.7)		1			1	
Agree	408 (51.1)	202 (34.3)	***<0.001***	***2.00***	***1.61–2.50***	***<0.001***	***1.76***	***1.39–2.24***
**Perceived severity**								
Complications of COVID-19 are very serious								
Disagree	247 (31.0)	336 (57.0)		1			1	
Agree	551 (69.0)	253 (43.0)	***<0.001***	***2.96***	***2.37–3.70***	***<0.001***	***2.68***	***2.11–3.41***
Will be very sick if I get COVID-19								
Disagree	533 (66.8)	482 (81.9)		1			1	
Agree	265 (33.2)	107 (18.2)	***<0.001***	***2.24***	***1.73–2.89***	***<0.001***	***2.02***	***1.53–2.68***
**Perceived benefits**								
Vaccination will decrease my chances of getting COVID-19								
Disagree	92 (11.5)	380 (64.5)		1			1	
Agree	706 (88.5)	209 (35.5)	***<0.001***	***13.1***	***10.6–18.4***	***<0.001***	***16.3***	***11.2–22.2***
**Perceived barriers**								
Concerned about the efficacy of the vaccination available								
Disagree	483 (60.5)	119 (20.2)		1			1	
Agree	315 (39.5)	470 (79.8)	***<0.001***	***0.17***	***0.13–0.21***	***<0.001***	***0.12***	***0.09–0.16***
Concerned about the safety/side effects of the vaccination available								
Disagree	407 (51.0)	80 (13.6)		1			1	
Agree	391 (49.0)	509 (96.4)	***<0.001***	***0.15***	***0.11–0.20***	***<0.001***	***0.13***	***0.09–0.17***
Concerned about the halal nature of the vaccination available								
Disagree	698 (87.5)	459 (77.9)		1			1	
Agree	100 (12.5)	130 (22.1)	***<0.001***	***0.51***	***0.38–0.67***	***<0.001***	***0.29***	***0.20–0.42***
**Cues to action**								
Will get vaccine after receiving complete information								
Disagree	133 (16.7)	229 (38.9)		1			1	
Agree	665 (83.3)	360 (61.1)	***<0.001***	***3.18***	***2.48–4.08***	***<0.001***	***2.77***	***2.12–3.60***
Will get vaccine if it is received by many in the public								
Disagree	493 (61.8)	447 (75.9)		1			1	
Agree	305 (38.2)	142 (24.1)	***<0.001***	***1.95***	***1.54–2.47***	***<0.001***	***1.82***	***1.41–2.34***
Will get vaccine if that does not cause undue problems to vaccinated people								
Disagree	358 (44.9)	159 (27.0)		1			1	
Agree	440 (55.1)	430 (73.0)	***<0.001***	***0.45***	***0.36–0.57***	***<0.001***	***0.43***	***0.34–0.55***

OR: Odds Ratio, AOR: Adjusted Odds Ratio, 95% CIs: 95% Confidence Intervals. Adjusted for: Gender, age, ethnicity, regions, education, occupation, chronic disease, diagnosis of COVID-19, receipt of flu vaccine.

## Data Availability

Data used for this study are presented in the manuscript and any request for raw data access will be available from the corresponding author upon reasonable request.
